# White matter and neurodevelopmental disorders: honoring Jean De Vellis through the work of the NICHD-funded intellectual and developmental disabilities research centers

**DOI:** 10.1186/s11689-019-9299-4

**Published:** 2019-12-16

**Authors:** Heather Cody Hazlett, Vittorio Gallo

**Affiliations:** 1 Department of Psychiatry, School of Medicine, UNC-Chapel Hill and Carolina Institute for Developmental Disabilities, Chapel Hill, NC USA; 2 Center for Neuroscience Research, Children’s National Research Institute, Children’s National Hospital, Washington, DC USA

Following in the footsteps of the first JND special issue in 2018, highlighting the work of the Eunice Kennedy Shriver Intellectual and Developmental Disabilities Research Centers (IDDRCs), this year again showcases the work of the IDDRCs and honors the distinguished career of Dr. Jean de Vellis, world-renowned scientist in the study of white matter pathophysiology and long-standing director of the UCLA IDDRC. As eloquently described in the dedication [1] accompanying this special section of the Journal, Jean de Vellis devoted his life’s work to examining glia biology and the importance of these neural cells in the brain. In this issue, we present cutting-edge, clinical and pre-clinical research and reviews by IDDRC investigators as a fitting tribute to the memory of our colleague.

Much of the promise in studying white matter comes from the potential this neural structure has for developing new treatments. In a review of both mouse models and clinical work [2], the novel approach of targeting glia cells to treat seizures in Tuberous Sclerosis Complex (TSC) may prove to be a successful treatment for these patients. Investigators at the Harvard-Boston Children’s IDDRC have demonstrated reduced white matter integrity in key fiber bundles may help to identify those individuals with TSC who also present with an autism spectrum disorder [3]. These investigators propose that widespread neuropathology of white matter may underlie the presence of autism in TSC. Similar findings of white matter abnormalities have been reported in autism spectrum disorder (ASD). Work at the UC Davis IDDRC suggests that white matter microstructure develops differently in young children with ASD, and in particular sex may play an important role in modulating the ASD neuroanatomical phenotype [4].

In addition to investigations of white matter morphology, a key aspect of thinking about novel treatments has come from thinking about inflammation and the negative impact this may have on neurodevelopment. Investigators at the Children’s Hospital of Philadelphia IDDRC present exciting work demonstrating that inflammation caused by Th2 cytokines during early brain development can be rescued with the application of IL-4 antibody treatment in the newborn rat [5]. This work highlights the importance of the immune system and impact on white matter development. This mechanism may play a role in injury and disorders that damage myelin, such as premature brain injury and cerebral palsy. In Alexander disease, a rare leukodystrophy resulting from demyelination, it is the accumulation of proteins that may first signify white matter abnormalities. The potential to design treatments targeting the accumulations of glial fibrillary acidic proteins (GFAP) is the focus of a review [6] by the investigators at the Waisman IDDRC, who are working to identify the toxicity of GFAP in these patients. Thinking more broadly about leukodystrophies, the team at the Kennedy Krieger IDDRC have sought to characterize a group of patients with a similar etiology [7]. Patients with mt-aaRS mutations have similar neurophenotypes, specifically observable abnormalities in white matter tracts on MRI.

As may be evident by work focusing on these neurodevelopmental disorders, a key benefit of approaching treatment stems from an ability to change developmental trajectories by acting early through specific interventions. In a comprehensive study examining motor neurons in a Down syndrome mouse model (Ts65Dn), the investigators demonstrated clear changes in the spinal white matter composition across the lifespan [8]. This work highlights the fluctuations in cellular properties, depending on developmental stage, which may help inform potential treatment target windows. The prospect of using white matter properties as a biomarker is further explored in a review of literature informed by ongoing work in the field of autism and related neurogenetic disorders such as fragile X syndrome [9]. A study of language disorder suggests where the field could be headed, where white matter biomarkers could be used to help monitor treatment effectiveness. Investigators at the Vanderbilt IDDRC found an important white matter tract known to be associated with language development, the inferior longitudinal fasciculus (ILF), showed differences in language outcomes [10].

The articles included in this special issue highlight the advances made in understanding white matter in neurodevelopmental disorders that investigators across the IDDRCs strive to tackle daily. By approaching these questions from both clinical (human) and basic neuroscience perspectives, both sides gain knowledge and move forward to the goal of better interventions. We hope that the work showcased in this special issue underscores the promise for developing targeted treatments in neurodevelopmental disorders that comes from these investigations. Many of the IDDRCs contain researchers influenced and inspired by the groundbreaking work Jean de Vellis began. We hope that this special issue also serves to honor his legacy to the field.

## Data Availability

Not applicable.
